# The cost‐effectiveness and budgetary impact of a dolutegravir‐based regimen as first‐line treatment of HIV infection in India

**DOI:** 10.1002/jia2.25085

**Published:** 2018-03-30

**Authors:** Amy Zheng, Nagalingeswaran Kumarasamy, Mingshu Huang, A David Paltiel, Kenneth H Mayer, Bharat B Rewari, Rochelle P Walensky, Kenneth A Freedberg

**Affiliations:** ^1^ Medical Practice Evaluation Center Massachusetts General Hospital Boston MA USA; ^2^ Y. R. Gaitonde Centre for AIDS Research and Education Voluntary Health Services Chennai India; ^3^ Yale School of Public Health New Haven CT USA; ^4^ Harvard Medical School Boston MA USA; ^5^ The Fenway Institute Fenway Health Boston MA USA; ^6^ Beth Israel Deaconess Medical Center Boston MA USA; ^7^ HIV/STI/Hepatitis Unit, Department of Communicable Diseases, World Health Organization Regional Office for South‐East Asia New Delhi India; ^8^ Division of General Internal Medicine Massachusetts General Hospital Boston MA USA; ^9^ Division of Infectious Diseases Massachusetts General Hospital Boston MA USA; ^10^ Division of Infectious Diseases Brigham and Women's Hospital Boston MA USA; ^11^ Department of Health Policy and Management Harvard T.H.Chan School of Public Health Boston MA USA

**Keywords:** HIV, India, dolutegravir, ART, modelling, cost‐effectiveness

## Abstract

**Introduction:**

Dolutegravir (DTG)‐based antiretroviral therapy (ART) is recommended for first‐line HIV treatment in the US and Europe. Efavirenz (EFV)‐based regimens remain the standard of care (SOC) in India. We examined the clinical and economic impact of DTG‐based first‐line ART in the setting of India's recent guidelines change to treating all patients with HIV infection regardless of CD4 count.

**Methods:**

We used a microsimulation of HIV disease, the Cost‐Effectiveness of Preventing AIDS Complications (CEPAC)‐International model, to project outcomes in ART‐naive patients under two strategies: (1) *SOC*: EFV/tenofovir disoproxil fumarate (TDF)/lamivudine (3TC); and (2) *DTG*: DTG + TDF/3TC. Regimen‐specific inputs, including virologic suppression at 48 weeks (*SOC*: 82% vs. *DTG*: 90%) and annual costs ($98 vs. $102), were informed by clinical trial data and other sources and varied widely in sensitivity analysis. We compared incremental cost‐effectiveness ratios (ICERs), measured in $/year of life saved (YLS), to India's *per capita* gross domestic product ($1600 in 2015). We compared the budget impact and HIV transmission effects of the two strategies for the estimated 444,000 and 916,000 patients likely to initiate ART in India over the next 2 and 5 years.

**Results:**

Compared to *SOC*,*DTG* improved 5‐year survival from 76.7% to 83.0%, increased life expectancy from 22.0 to 24.8 years (14.0 to 15.5 years, discounted), averted 13,000 transmitted HIV infections over 5 years, increased discounted lifetime care costs from $3040 to $3240, and resulted in a lifetime ICER of $130/YLS, less than 10% of India's *per capita* GDP in 2015. *DTG* maintained an ICER below 50% of India's *per capita* GDP as long as the annual three‐drug regimen cost was ≤$180/year. Over a 2‐ or 5‐year horizon, total undiscounted outlays for HIV‐related care were virtually the same for both strategies.

**Conclusions:**

A generic DTG‐based regimen is likely to be cost‐effective and should be recommended for initial therapy of HIV infection in India.

## 1 Introduction

Dolutegravir (DTG), an integrase strand transfer inhibitor, is recommended as a first‐line antiretroviral drug for the treatment of HIV infection in the United States and Europe 1. Several studies have proven the non‐inferiority or superiority of DTG, with its more tolerable side effect profile, high genetic barrier to resistance, and decreased time to virologic suppression, compared to alternative first‐line antiretroviral agents (Table S1, Appendix S1) 2, 3. In addition to DTG's excellent clinical profile, it has also been shown to be an economically attractive strategy in both treatment‐naïve and treatment‐experienced patients in North America and Europe 4, 5, 6.

India is home to the world's third largest population of people living with HIV (PLHIV), with an estimated 2.1 million infected people 7. While the number of reported new HIV infections per year decreased by 66% from 130,000 in 2000 to 86,000 in 2015, more than 60% of Indian PLHIV remain untreated 7, 8. As of early 2017, an estimated 1,013,000 PLHIV in India are receiving first‐line ART and 13,000 people are on second‐line ART at government clinics supported by the National AIDS Control Organization (NACO) 9, personal communication with Dr. Bharat Bhushan Rewari]. Efavirenz (EFV) in combination with tenofovir disoproxil fumarate (TDF) and lamivudine (3TC) is the recommended first‐line ART regimen in India. Generic DTG is not yet widely available, although it was recently launched in India 10, 11. Given the improved virologic suppression and fewer side effects of DTG‐ compared to EFV‐based regimens, implementation of DTG‐based first‐line ART could substantially reduce the number of people who fail first‐line treatment and are lost to care or require a switch to second‐line ART 2, 3.

With the rollout of universal antiretroviral treatment in May 2017, cost will be an increasingly central consideration in future ART recommendations to ensure that treatment can be provided to the current population of PLHIV who are newly eligible for treatment as well as to those with new HIV diagnoses 12. While the current cost of co‐formulated EFV/TDF/3TC in India is $98 USD/year, recent Clinton Health Access Initiative (CHAI) cost estimates for a DTG‐based regimen were $102 USD/year 13.

With generic DTG becoming increasingly available from multiple Indian pharmaceutical companies 14, we used simulation modelling to examine the potential cost‐effectiveness and budgetary impact of a DTG‐based first‐line ART strategy in India.

## 2 Methods

### 2.1 Analytic overview

We used the Cost‐Effectiveness of Preventing AIDS Complications‐International (CEPAC‐I) model 15, 16, a microsimulation model of HIV disease and treatment, to project and compare the clinical and economic performance of EFV/TDF/3TC, the standard of care (hereafter *SOC*), to DTG+TDF/3TC (hereafter *DTG*) for treatment‐naïve, HIV‐infected patients initiating ART in India.

Clinical and economic model outcomes include life expectancy, proportion of people alive, proportion of people remaining on first‐line ART, number of HIV infection transmissions averted, and both cumulative ART and non‐ART costs of HIV care; we assessed these outcomes at 2‐year, 5‐year, and lifetime horizons. We calculated incremental cost‐effectiveness ratios (ICERs) for each strategy using life expectancy and lifetime costs, both discounted at 3% per year, in 2016 USD 17. This discount rate functions as a median value between estimated discount rates across different regions in India 18.

We defined a strategy as “cost‐effective” if its incremental cost per year‐of‐life saved (YLS) was less than $800, 50% of the 2015 Indian *per capita* GDP, as recommended by Woods et al. for low to middle‐income countries 19, 20. We also conducted a budget impact analysis (BIA, undiscounted by convention 21) of a DTG‐based first‐line ART regimen at 2 and 5 years over a range of DTG regimen costs. As there are no published clinical trial data on the performance of DTG in resource‐limited settings, we performed sensitivity analysis on the clinical efficacy of DTG to assess the impact of relative drug efficacy on the results.

### 2.2 The cost‐effectiveness of preventing AIDS complications‐international (CEPAC‐I) model

The CEPAC‐I model is a patient‐level microsimulation of HIV disease, treatment, and associated care costs in resource‐limited settings 15, 16. Simulated HIV‐infected patients have characteristics drawn randomly from model user‐defined distributions of age, sex, CD4 count at treatment initiation, and plasma HIV RNA level. The cohort undergoes monthly transition probabilities between health states that simulate a unique disease and treatment trajectory for each individual patient. Clinical events and treatment costs are projected over each patient's lifetime. To achieve stable per‐person estimates for different strategies, cohorts of one million patients are simulated. A complete technical specification of the CEPAC‐I model can be found at http://www.massgeneral.org/mpec/cepac/.

In accordance with the recently updated HIV treatment guidelines of India's NACO, all HIV‐infected patients are eligible to initiate ART upon presentation to care 10, 12. The efficacy of ART is dependent upon each patient's adherence level, an attribute assigned via a random draw from a logit‐normal distribution (0 to 100%); patients who are more adherent have a higher likelihood of virologic suppression (HIV RNA <50 copies/mL) at 48 weeks and are more likely to remain in HIV care 22. After 48 weeks, patients on a suppressive ART regimen face a monthly probability of “late” virologic failure (HIV RNA > 5000 copies/mL 10) similarly dependent on their adherence. Patients on ART are immunologically monitored every 6 months, and treatment failure is detected by either a new or recurrent Stage 4 opportunistic disease (OD) after at least 6 months on treatment or a declining CD4 count. Patients do not receive regular viral load monitoring; however, regimen failure is confirmed via a viral load test (in addition to regular CD4 monitoring), as per current practice in India 10. Patients who fail first‐line ART, presumably due to poor adherence, are given the opportunity to achieve suppression on first‐line ART one more time prior to becoming eligible to receive a protease‐inhibitor (PI)‐based second‐line ART regimen; they are again subject to adherence level‐dependent rates of virologic suppression and late failure 10.

### 2.3 Model input parameters

#### 2.3.1 Cohort characteristics

The simulated cohort reflected the demographic and clinical characteristics of untreated HIV‐infected patients initiating ART in India (Table 1) 23. The cohort was 57% male with a mean (SD) age of 37 (8) years, and a mean (SD) CD4 count of 192 (109) cells/μL at the time of ART initiation 23. Loss to follow‐up rates were informed by data on Indian patients in the national ART programme 24, 25. Patients who are lost do not accrue care costs but continue to contribute to overall cohort mortality.

**Table 1:** Base case input parameters for a model‐based analysis of DTG‐based first‐line ART in India

Parameter	EFV/TDF/3TC	DTG + TDF/3TC [range assessed]	Reference
Cohort characteristics			
Gender, % male	57		
Age, years, mean (SD)	37 (8)		23
CD4 count at presentation, cells/μL, mean (SD)	192 (109)		
Baseline ART Adherence, %			
Adherence ≤50%	7		
Adherence 50 to 95%	57		22
Adherence ≥95%	37		
ART efficacy			
1st‐line overall suppression at 48 weeks, %	82	90 [79 to 96]	3
Re‐treatment suppression, %	19	19	23
Virologic failure for suppressed patients, %/month	0.32	0.21 [0 to 1.0]	3, 26, 27
Monthly CD4 Increase on ART, cells/μL			
First month, mean (SD)	83 (38)	107 (30) [80 to 134]	3
After first month, mean (SD)	4 (2)	5 (2)	
ART toxicity			
Nephrotoxicity due to TDF			
Probability, %/month	1.0 [1.0 to 2.0]		48
Months to toxicity, mean	5		
Retention in care			
Loss to follow‐up, %/month			
Adherence <50%	1.6		24, 25
Adherence >95%	0.2		
HIV Infection Transmission (per person/month), rate/100PY			
On ART	0.46		32
HIV RNA level, copies/mL			
>100,000	9.03		
10,001 to 100,000	8.12		
3001 to 10,000	4.17		32
501 to 3000	2.06		
≤500	0.16		
Annual costs, 2016 US $			
1st‐line ART	98	102 [60 to 300]	13
2nd‐line ART (PI‐based regimen)	246 [98 to 318]		

DTG, dolutegravir; ART, antiretroviral therapy; EFV, efavirenz; TDF, tenofovir disoproxil fumarate; 3TC, lamivudine; SD, standard deviation; PY, person‐year; PI, protease inhibitor.

#### 2.3.2 ART regimens, efficacy and costs

In the model, ART‐eligible patients are started on either *SOC*, an EFV‐based regimen that is the current standard of care in India, or *DTG* (DTG + TDF/3TC) as first‐line therapy. Although the SINGLE trial compared DTG + abacavir (ABC)/3TC (and not DTG + TDF/3TC) to EFV/TDF/3TC, we used data from this trial to project the efficacy of DTG+TDF/3TC, recognizing that screening for the HLA‐B*5701 allele is not routinely available, and therefore regimens containing abacavir are not likely to be used in an Indian setting 10. Based on findings from the US‐based SINGLE trial, patients on *SOC* experienced an overall virologic suppression rate of 82% and those on the comparator *DTG* strategy an overall suppression rate of 90%, both at 48 weeks 3. These rates omit those who were lost to follow‐up, withdrew consent, or switched regimens for other reasons in the trial (these events are captured in other parameters of the model) and therefore do not reflect full intent‐to‐treat values; consequently, our estimated inputs for virologic suppression rates are likely higher than what would be observed in an Indian cohort. In the 2 months following ART initiation, patients experienced a monthly mean (SD) CD4 count increase of 83 (38) on *SOC* and 107 (30) cells/μL on *DTG*, with lower but sustained CD4 count increases thereafter 3. After 48 weeks on treatment, patients on suppressive *SOC* and *DTG* became subject to monthly probabilities of late virologic failure of 0.32% and 0.21%, as derived from the SINGLE trial at 96 weeks 3, 26, 27. As failure on *DTG* was derived from that of a DTG + ABC/3TC regimen, it may be an overestimate, given reported failure rate differences in regimens containing ABC/3TC and TDF/FTC 28. However, we used this failure rate to maintain a conservative modelling approach. Patients confirmed to have virologically failed first‐line therapy and have had a one‐time opportunity for re‐suppression were then switched to a subsequent and last PI‐based ART regimen of atazanavir (ATV)/ritonavir/TDF/3TC 10, 29.

We derived ART regimen costs from HIV drug prices in resource‐limited countries published by CHAI 13; we assigned annual per‐person three‐drug regimen costs of $98 to *SOC*, $102 to *DTG*, and $246 to second‐line PI‐based therapy. We assumed that switching to a DTG‐based regimen would not result in a substantial change to the supply chain, since DTG does not need to be refrigerated and is likely to be available in fixed‐dose combination 30. We estimated routine care costs from an economic analysis of in‐patient and out‐patient services provided by YRG CARE 31. All costs are reported in 2016 US dollars.

#### 2.3.3 HIV transmissions

We projected the cumulative number of incident HIV infections transmitted over 5 years by two populations of PLHIV: (1) patients who newly present to care and are immediately eligible to initiate ART, irrespective of CD4 count (n = 125,000 each year); and (2) patients currently engaged in care with CD4 counts from 350 to 500/μL (n = 120,000 total; 40,000/year assumed to initiate ART over next 3 years) and >500/μL (n = 170,000 total; 57,000/year assumed to initiate ART over next 3 years) who are expected to initiate ART as a result of recent changes to ART eligibility criteria to treat all infected regardless of CD4 count (n = 916,000 in total) 9, personal communication with Dr. Bharat Bhushan Rewari]. For a more detailed explanation of ART uptake, please see Table S2 in the Appendix S1.

To project the number of HIV infections transmitted per person per month over 5 years by those newly initiating care under both *SOC* and *DTG*, we used model‐generated HIV RNA output and published meta‐analysis‐based estimates of HIV transmission risk in heterosexual, serodiscordant couples in which the seropositive partner is on ART, ranging from 0.16 to 9.03 transmissions/100 person‐years (PY), depending on HIV RNA level (Table 1) 32. To calculate the total number of HIV infections transmitted by the entire population of PLHIV on ART, we used a meta‐analysis‐based estimate of HIV transmission risk in patients on ART (Table 1) 32. In addition, we determined the expected cost‐savings over the next 5 years as a result of reduced transmissions from those newly initiating ART (see Table S2 and Figure S1 in Appendix S1 for methods and calculations).

#### 2.3.4 One‐way and multi‐way sensitivity analysis

Given uncertainties around both clinical and cost parameters, we conducted one‐way sensitivity analyses on multiple parameters of *DTG*, including: 48‐week suppression rate (79 to 96%); monthly probability of virologic failure on suppressive *DTG* after 48 weeks (0 to 1.0%); monthly CD4 count change on *DTG* (multiplier of 0.75 to 1.25); monthly probability of nephrotoxicity due to TDF in both *SOC* and *DTG* (1.0 to 2.0%); annual three‐drug regimen cost of *DTG* ($60 to 300/person); and annual regimen cost of second‐line ART ($98 to 318/person). Based on the results of the one‐way sensitivity analysis, we then subjected the most influential parameters to additional multi‐way sensitivity analysis.

#### 2.3.5 Budget impact analysis

To assess the fiscal consequences to payers of implementing a DTG‐based regimen as first‐line ART on the national HIV programme in India, we conducted a BIA of actual outlays over both 2 and 5 years. We projected the cumulative outlays for HIV care over the two time periods for the same two populations of PLHIV outlined above: (1) patients who newly present to care and are immediately eligible to initiate ART; and (2) patients currently engaged in care with CD4 counts >350/μL 9, personal communication with Dr. Bharat Bhushan Rewari]. This leads to a total of 444,000 and 916,000 patients initiating ART over 2 and 5 years. This analysis accounted for differences in annual first‐line ART costs, including those for possible efforts at re‐suppression, second‐line ART costs for those who fail first‐line therapy, and associated non‐ART medical care costs, including monitoring tests and routine care.

## 3 Results

### 3.1 Clinical outcomes

Compared to *SOC*, survival under *DTG* increased from 86.7% to 90.2% at 2 years and 76.7% to 83.0% at 5 years (Table 2). Undiscounted life expectancy was also higher under *DTG* compared to *SOC* (24.8 vs. 22.0 years; discounted [3%/year]: 15.5 vs. 14.0 years). In addition, a greater proportion of treated HIV‐infected patients remained on first‐line therapy with *DTG* compared to *SOC* at 5 years (99.2% vs. 97.9%).

**Table 2:** Base case clinical and economic model outcomes of strategies comparing a DTG‐based first‐line ART regimen with an EFV‐based first‐line ART regimen in India

	Undiscounted results							Discounted^a^ results		
Strategy	Proportion of patients alive at 2 years (%)	Proportion of patients alive at 5 years (%)	Life Expectancy (YLS)	Proportion of patients on first‐line ART^b^ at 2 years (%)	Proportion of patients on first‐line ART at 5 years (%)	2‐year programme cost^c^ (2016 USD, millions)	5‐year programme cost^c^ (2016 USD, millions)	Lifetime per person cost (2016 USD)	Life Expectancy (YLS)	ICER^d^($/YLS)
EFV/TDF/3TC (*SOC*)	86.7	76.7	22.0	99.8	97.9	139	590	3,040	14.0	‐
DTG+TDF/3TC	90.2	83.0	24.8	99.9	99.2	137	590	3,240	15.5	130

DTG, dolutegravir; EFV, efavirenz; ART, antiretroviral therapy; TDF, tenofovir disoproxil fumarate; 3TC, lamivudine; SOC, standard of care; USD, US dollars; YLS, year‐of‐life saved; ICER, incremental cost‐effectiveness ratio.

a Discounted at a rate of 3%/year.

b Proportion of patients on first‐line ART out of all patients alive and on ART.

c Costs projected for cohorts of 444,000 and 916,000 ART‐eligible patients initiating treatment over 2 and 5 years.

d ICER calculated using exact numbers and rounded to nearest ten.

### 3.2 HIV transmissions

Based on 914,000 people presenting to care and initiating ART over the next 5 years, an estimated 57,400 HIV transmissions are expected in 5 years under *SOC*, compared to 44,400 transmissions with *DTG* as first‐line therapy—a 23% decrease in incident infections. An additional 23,600 HIV transmissions are expected in 5 years from the population of PLHIV who are currently in care and on ART (1,013,000 and 13,000 patients on first‐line and second‐line ART), assuming no treatment switch in those patients 9, personal communication with Dr. Bharat Bhushan Rewari]. The reduction in transmissions from those starting ART, when considered with the improved effectiveness of *DTG* compared to *SOC*, will save an estimated additional $800,000 over the next 5 years. These cost‐savings encompass the care costs associated with all second‐generation infections, including transmissions averted in the case of *DTG* (Figure S1 in Appendix S1). Further cost‐savings will be realized in the long‐term as more newly‐infected patients enter care.

### 3.3 Cost and cost‐effectiveness outcomes

Discounted per‐person lifetime costs were $3040 for *SOC* and $3240 for *DTG* (Table 2). At an annual regimen cost of $102, *DTG* was cost‐effective compared to *SOC* with an ICER of $130/YLS—less than 10% of India's annual *per capita* GDP.

### 3.4 One‐way sensitivity analysis

**Figure 1:**
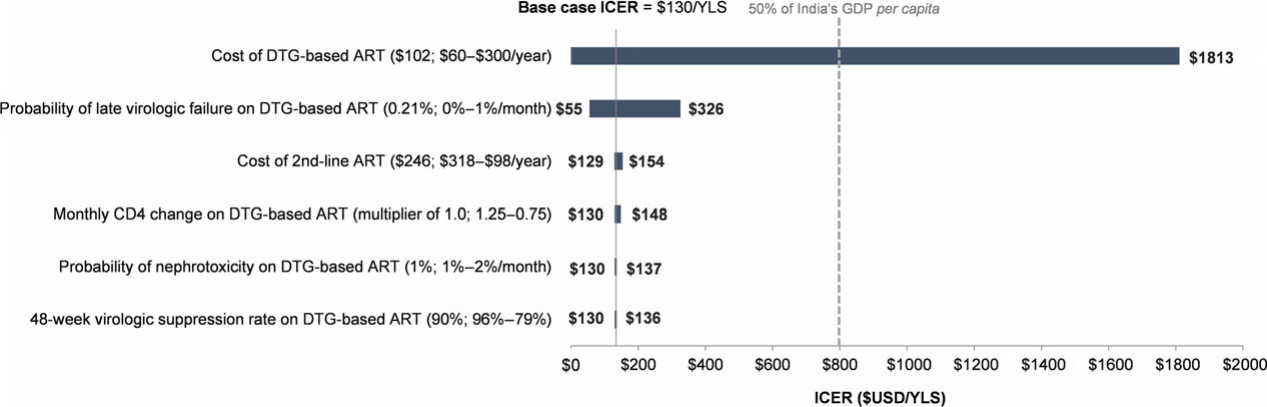
One‐way sensitivity analysis on the cost‐effectiveness of *DTG* compared to *SOC*. The horizontal bars represent the range of ICERs obtained when varying a single model parameter across its plausible range. Ranges examined are presented next to the parameter label as (base case input; parameter input that confers the lowest ICER – parameter input that confers the highest ICER). Parameters are arranged along the vertical axis in order of their impact on the ICER, with the most influential parameters at the top of the axis. The grey dashed line represents 50% of the Indian annual *per capita* GDP in 2015 ($800). ICERs below 50% of the *per capita* GDP are considered cost‐effective. DTG, dolutegravir; ART, antiretroviral therapy; USD, US Dollars; ICER, incremental cost‐effectiveness ratio; GDP, gross domestic product; YLS, year‐of‐life saved.

When we varied each parameter across its plausible range, holding all other parameters at their base case values, we found that the two most influential parameters were the annual cost of the DTG regimen and the monthly probability of late virologic failure (after 48 weeks on suppressive *DTG*, Figure 1).

### 3.5 Multi‐way sensitivity analysis

**Figure 2:**
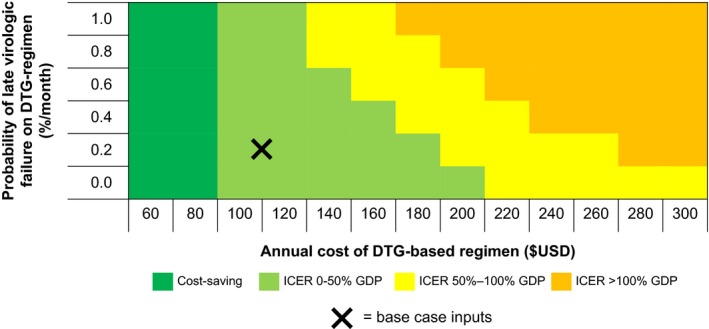
Multi‐way sensitivity analysis on the cost‐effectiveness of *DTG* compared to *SOC* in India. The figure reports changes in the incremental cost‐effectiveness ratio (ICER) of *DTG* compared to *SOC* when simultaneously varying the annual cost of *DTG* and the monthly probability of virologic failure after 48 weeks on *DTG*. The horizontal axis denotes the range of annual costs of *DTG*. The vertical axis denotes the range of monthly probabilities of late failure for those virologically suppressed on the DTG regimen. The black “X” marks the characteristics of the base case *DTG*. The colours of the cells represent ICER categorization, ranging from “not cost‐effective” (i.e. *DTG* confers a greater number of life years than *SOC* but at an incremental cost per life‐year that exceeds 50% of the Indian *per capita* GDP, yellow [51 to 100% of *per capita* GDP] and orange [greater than 101% of *per capita* GDP] cells), “cost‐effective” (i.e. *DTG* confers a greater number of life years than *SOC* at an incremental cost per life‐year that is less than the national *per capita* GDP, light green [0 to 50% of the *per capita* GDP] cells), to “cost‐saving” (i.e. *DTG* both costs less and confers a greater number of life‐years than *SOC*, dark green cells). DTG: dolutegravir. ART, antiretroviral therapy; USD, US Dollars; ICER, incremental cost‐effectiveness ratio; GDP, gross domestic product.

We then considered simultaneous variation in the two most influential parameters identified above. At a regimen cost less than $80 per‐person per year, *DTG* was cost‐saving across examined values of late virologic failure. At a three‐drug regimen cost of $102 per‐person per year, *DTG* was cost‐effective, with an ICER less than 50% of *per capita* GDP, across the assessed range of late virologic failure probabilities (Figure 2). Even at an annual drug cost more than twice that of *SOC* ($200), *DTG* remained cost‐effective with an ICER less than 50% of *per capita* GDP, as long as the late virologic failure probability was less than 0.2%/month. At *DTG*'s expected late virologic failure rate (0.21%/month), the regimen's ICER remained below 50% of the Indian *per capita* GDP so long as its annual regimen cost ≤$180/year. When we varied second‐line ART costs simultaneously in both strategies, *DTG* remained cost‐effective when compared to *SOC* (Figure S2 in Appendix S1).

### 3.6 Budget impact analysis

**Figure 3:**
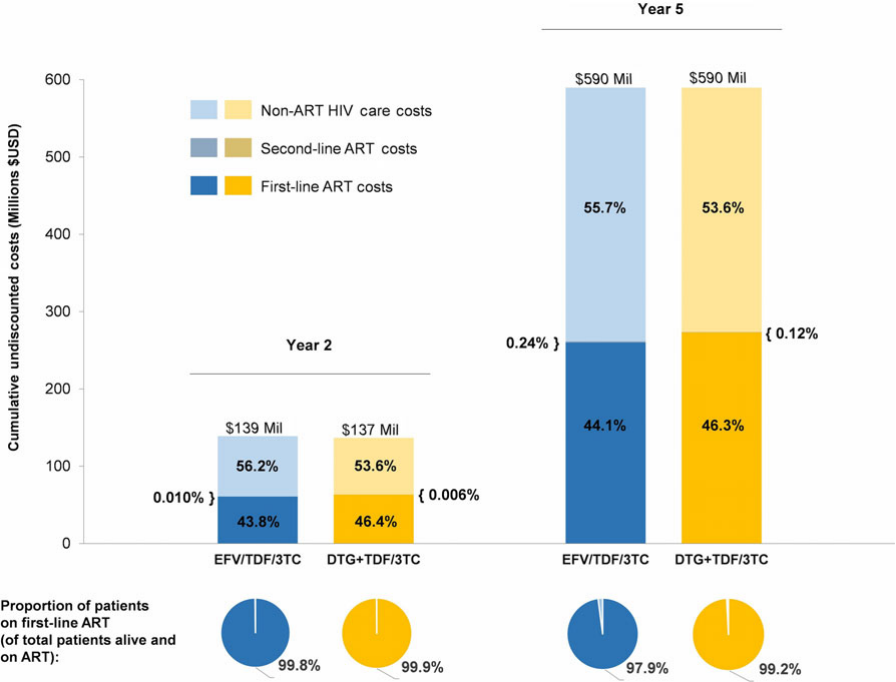
Budget impact analysis and proportion of patients on first‐line ART at 2 and 5 years for *SOC* and *DTG* in India. Total undiscounted costs of care at 2 and 5 years after ART initiation for a cohort initiating HIV care stratified into three categories: first‐line ART costs (dark blue/yellow), second‐line ART costs (hatched blue/hatched yellow), and non‐ART HIV care costs (light blue/yellow). We projected the cumulative costs of HIV care over the two time points for two populations living with HIV: (1) patients who newly present to care and are eligible to initiate ART (n = 125,000 each year); (2) patients currently in care with CD4 counts between 350 and 500/μL (n = 120,000 total; 40,000/year assumed to initiate ART over next 3 years) and >500/μL (n = 170,000 total; 57,000/year assumed to initiate ART over next 3 years) who are expected to initiate ART as a result of recent changes to ART eligibility criteria to treat all infected regardless of CD4 count (n = 444,000 over 2 years and 916,000 over 5 years). We assume annual costs of *SOC* and *DTG* to be $98 and $102. The percentage of each cost category as a proportion of total HIV care costs is labeled in each bar. The percentage of patients on first‐line ART as a proportion of patients who are alive and on ART at the end of years two and five are shown below each bar. TDF, tenofovir disoproxil fumarate; 3TC, lamivudine; EFV, efavirenz; DTG, dolutegravir; ART, antiretroviral therapy; USD, US Dollars; Mil, million.

Cumulative undiscounted medical care costs for the estimated 444,000 and 916,000 treatment‐naïve, HIV‐infected patients initiating *SOC* are estimated to be $139 million at 2 years and $590 million at 5 years. Compared with *SOC*, the cost at 2 years is lower ($137 million) and cost‐neutral at 5 years ($590 million) under *DTG* (Figure 3). Higher proportions of patients were alive and remained on first‐line ART with *DTG* as time proceeds: 99.9% vs. 99.8% at 2 years and 99.2% vs. 97.9% at 5 years—an improvement in clinical outcomes at no additional cost to the national programme.

With more patients on second‐line ART under *SOC*, 0.24% of cumulative 5‐year care costs were attributable to second‐line ART compared to 0.12% for *DTG*.

If annual drug costs are lower than anticipated in the base case, cost savings would be more pronounced. At an annual three‐drug regimen cost of $75 per‐person 33, the budget for *DTG* would be $120 million at 2 years and $518 million at 5 years, making it cost‐saving compared to *SOC* by $19 and $72 million at the 2‐ and 5‐year mark, respectively.

An annual three‐drug regimen cost of $105 per‐person was the threshold at which *DTG* became cost‐neutral at 2 years ($139 million) compared to *SOC*; costs higher than this threshold led to increases in budget at both 2 and 5 years. The 2‐ and 5‐year budget impact over a range of DTG regimen costs can be found in Table S3 of Appendix S1.

## 4 Discussion

The impressive clinical profile of dolutegravir‐based ART, its widespread use in the United States and Europe, and its inclusion as first‐line therapy in Botswana and Kenya, indicate its potential to become a WHO‐recommended and preferred first‐line regimen for HIV‐infected populations in low‐ and middle‐income countries, rather than its current status as an alternative first‐line option in the 2016 guidelines 33, 34, 35. Given the increasingly constrained healthcare funds allocated to AIDS programmes in the setting of concurrent expansion of HIV treatment eligibility criteria in India, ART cost remains critically important in the context of HIV guidelines there, as in other countries 36. With the rollout of generic dolutegravir from Indian drug‐makers at $75 per‐patient per year in African countries 35, 37, as well as the availability of generic formulations in India's private health sector as of March 2017 38, the use of generic DTG in the national programme is a near‐term consideration for the Indian government.

Using a mathematical simulation model of HIV disease and treatment, we found that the implementation of a DTG regimen for initial treatment of HIV infection was demonstrably cost‐effective, and potentially cost‐saving from the health systems perspective, across a wide range of plausible assumptions regarding ART efficacy, virologic failure rates after 48 weeks on ART, and drug costs**.** Over 2‐ and 5‐year time horizons, a greater proportion of treated HIV‐infected patients remained on first‐line ART with *DTG* as initial treatment, compared to *SOC*. At an ART cost close to the current cost of *SOC*, as currently proposed by the Clinton Health Access Initiative, a DTG regimen provided substantial clinical benefits at little to no additional cost to the programme. Any potential cost increase reflected longer life expectancy, and thus greater lifetime care costs for HIV‐infected persons. Furthermore, these costs did not fully capture the clinical benefit and potential cost savings of averting 13,000 incident HIV infections through more effective treatment, a greater than 20% decrease in HIV incidence over 5 years. Due to the robustness of our results across a wide range of parameter variation for DTG‐based regimen suppression rates, our conclusions are not specific to a randomized, trial based setting and instead capture a range of possible outcomes, suggesting both cost‐efficacy and cost‐effectiveness.

The potential savings associated with implementing DTG‐based first‐line therapy are driven primarily by the smaller number of patients experiencing virologic failure on the DTG compared to SOC regimen and needing to be switched to more costly protease inhibitor‐based second‐line ART. Given the lack of readily available third‐line ART and drug resistance testing after virologic failure in India, a DTG regimen, with its high barrier to resistance, would be a compelling alternative to the current standard of care 39.

As per current practice in India, our base case inputs did not include regular viral load (VL) monitoring. However, the Indian government has recently changed its guidelines to recommend yearly VL monitoring for patients on first‐line ART 40. Therefore, we conducted an additional sensitivity analysis in which VL monitoring was included in both *SOC* and *DTG*. While increasing the overall budget impact in both strategies, VL monitoring marginally improved the ICER of *DTG* compared to *SOC* to $100/YLS, as it helped identify virologic failure sooner in *SOC*, causing even more patients to switch to costly second‐line ART.

It is also estimated that in India—the country with the highest TB burden in the world—nearly 20% of HIV‐infected people are co‐infected with TB 41. The viability of DTG‐based ART as initial treatment in India is dependent upon DTG's lack of drug‐drug interactions with rifamycin 42. Promising results of a recent study demonstrate that the co‐administration of twice‐daily DTG with rifamycin or once‐daily DTG with rifabutin is both safe and effective 43, and an open label clinical trial is underway to compare DTG with EFV‐based ART regimens for the treatment of HIV‐TB co‐infection 44. Should a DTG‐based regimen prove superior, or non‐inferior, to an EFV‐based ART regimen in co‐infected patients, this would provide additional justification for its inclusion as first‐line ART.

This analysis has several limitations. There are currently no efficacy data directly comparing DTG + TDF/3TC to EFV/TDF/3TC, though the results of a direct comparison trial (ADVANCE) are expected in 2018 45. We therefore assessed a DTG‐based regimen based on the clinical profile of DTG + ABC/3TC, as reported in the US‐based SINGLE trial, though regimens containing abacavir would not be used in an Indian setting given that HLA testing is not standard of care 10. We also recognize that the late virologic failure rate of the DTG‐based regimen may even be an overestimate, given reported failure rate differences in regimens containing ABC/3TC and TDF/FTC 28. Furthermore, as resistance testing is not readily available, we did not include resistance to EFV in our simulation despite a reported resistance of 11% to non‐nucleoside reverse transcriptase inhibitors among treatment‐naïve patients in India 46. In so far as some of these patients would not achieve virologic suppression on a first‐line EFV‐based regimen, this would make the DTG regimen look even more attractive. The analysis was also limited to the cost‐effectiveness of starting ART‐naïve patients on a DTG regimen and did not account for either the potential clinical benefits or costs of those who switch from an EFV‐based regimen to DTG. If those already on EFV‐based regimens were also switched, there would be some additional clinical benefit from fewer late virologic failures as well as some additional transmissions prevented. The cost impact of switching these additional patients would depend on the relative costs of the two regimens. We also used historical numbers of ART‐naïve HIV‐infected patients who initiated therapy between March 2014 and March 2015, as well as estimates of those with CD4 counts 350 to 500/μL and >500/μL who became ART‐eligible in 2015 and 2017, to calculate the 2‐ and 5‐year budget impact of a DTG regimen 9, personal communication with Dr. Bharat Bhushan Rewari]; it is possible that these numbers will increase in the future with greater availability of ART 12. In accounting for the number of incident HIV infections averted due to patients initiating DTG‐based ART, we used transmission rates derived from studies across multiple countries, including India, that assessed HIV infections transmitted among serodiscordant, heterosexual couples; this may not necessarily reflect the true risk of HIV transmission in India, where the epidemic is primarily concentrated among persons who inject drugs, men who have sex with men, and transgender people 9. Moreover, unit cost data for non‐ART patient care are consistent with the most recent published HIV care costs 47. Sensitivity analysis around these costs lead to consistent ICERs for a DTG‐based regimen of less than 50% of *per capita* GDP.

We did not explicitly factor disability‐adjusted life years into our analysis. However, given that *DTG* is highly cost‐effective and even cost‐saving in the short term compared to *SOC*, even if we conservatively assumed the average year of life gained had a disability weight of 0.5, *DTG* would still be in a range well below the 50% GDP threshold suggested by Woods et al. 21. A key feature of this policy choice is that switching to a DTG‐based regimen saves the health sector money in the short‐to‐medium term, as it reduces the need for more expensive second‐line therapy. In the long run, *DTG* may become more costly due to increased survival and additional years of HIV care required. The switch in policy from *SOC* to *DTG* is a less risky investment than many health interventions which require initial investment, with benefits only accruing in the long run.

## 5 Conclusions

In conclusion, we found that implementation of a DTG‐based regimen as first‐line therapy for HIV infection in India substantially improves overall survival, increases life expectancy, and is likely to be cost‐effective—potentially even cost‐saving—compared to the current standard of care EFV‐based regimen. When generic DTG becomes available in India, if priced no more than $180 per year, it should quickly be recommended for first‐line ART.

## Competing interests

The authors have no competing interests to declare.

## Authors’ contributions

AZ and KAF contributed to the conception and design of the work. NK, KHM and BBR contributed to the acquisition of data. AZ, KAF, MH, ADP, and RPW contributed to the analysis and interpretation of data for the work. All authors contributed substantially to drafting and revising the work critically for intellectual content and have approved the final version of the work to be published.

## Supporting information


**Appendix S1**. Additional results and sensitivity analyses with in‐depth technical information.
**Table S1.** Additional comparators of DTG and EFV‐based regimens from the SINGLE trial [3].
**Table S2.** Treatment uptake and scale up over the next five years.
**Table S3.** Two‐ and five‐year budgetary impact of a DTG‐based regimen across a range of annual costs.
**Figure S1.** Schematic diagram for calculating HIV transmissions over 5 years under *SOC* and *DTG*.
**Figure S2.** Multi‐way sensitivity analysis on the cost‐effectiveness of *DTG* compared to *SOC* in India while simultaneously varying *DTG* cost, probability of virologic failure with *DTG*, and second‐line ART cost.
