# Supplementary material for: The cost‐effectiveness and budgetary impact of a dolutegravir‐based regimen as first‐line treatment of HIV infection in India
Source: J Int AIDS Soc. 2018 Mar 30;21(3):e25085. doi: 10.1002/jia2.25085 (PMC5878415; doi:10.1002/jia2.25085)
Supplement: Supplementary file 1 — Appendix S1. Additional results and sensitivity analyses with in‐depth technical information. Table S1. Additional comparators of DTG and EFV‐based regimens from the SINGLE trial [3]. Table S2. Treatment uptake and scale up over the next five years. Table S3. Two‐ and five‐year budgetary impact of a DTG‐based regimen across a range of annual costs. Figure S1. Schematic diagram for calculating HIV transmissions over 5 years under SOC and DTG. Figure S2. Multi‐way sensitivity analysis on the cost‐effectiveness of DTG compared to SOC in India while simultaneously varying DTG cost, probability of virologic failure with DTG, and second‐line ART cost. [file JIA2-21-e25085-s001.docx]

**Supplementary Material:**

**The cost-effectiveness and budgetary impact of a dolutegravir-based regimen as first-line treatment of HIV infection in India**

Amy Zheng, BA

Nagalingeswaran Kumarasamy, MD, PhD

Mingshu Huang, MA, PhD

A. David Paltiel, PhD

Kenneth H. Mayer, MD

Bharat B. Rewari, MD

Rochelle P. Walensky, MD, MPH

Kenneth A. Freedberg, MD, MSc

This appendix provides supplementary information on the efficacy of DTG, treatment uptake, and HIV transmissions over 5 years, as well as additional sensitivity analyses on the annual cost of second-line ART and on the annual cost of a DTG-based ART regimen for the two- and five-year budget impact analysis.

* * *

**Table S1.** Additional comparators of DTG and EFV-based regimens from the SINGLE trial [3].

| **Parameter** | **Treatment Group** | |
| --- | --- | --- |
|  | DTG+ABC/3TC | EFV+TDF/FTC |
| Median time to viral suppression, days | 28 | 84 |
| Increase in CD4 count at 48 weeks, cells/µL | 267 | 208 |
| Patients who discontinued therapy due to adverse  events, % | 2 | 10 |

**DTG:** dolutegravir. **EFV:** efavirenz. **TDF:** tenofovir disoproxil fumarate. **3TC:** lamivudine.

**ABC:** abacavir.

**Table S2.** Treatment uptake and scale up over the next five years.

In our model, 84,000 patients who present with CD4 counts <350/µl will enter care each year. Moreover, each year, 18,000 and 23,000 new patients link to care with CD4 counts from 350-500/µl and >500/µl, respectively [[9](#_ENREF_9)].

However, Indian guidelines have recently changed to immediate ART initiation regardless of CD4 count at presentation. Starting in May 2017, additional patients with higher CD4 counts who are currently in care but not yet on ART (due to previous ineligibility) began treatment. The total population in care and newly eligible to start ART consists of approximately 120,000 people with CD4 counts from 350-500/µl and 170,000 people with CD4 counts >500/µl [personal communication with Dr. Bharat Bhushan Rewari]. We assumed that a third of each group would be able to initiate ART every year. This rate of initiation is likely feasible in this context, since the number of ART centers increased by almost two-fold from 2010 to the end of 2015, with the number of people living with HIV on ART increasing from 316,000 to 903,000 over that period [9].

These numbers mirror the current pace of HIV detection in India and are only reflective of changing guidelines for ART initiation, and thus, no further budgeting for HIV testing would be required.

| ***CD4 group*** | **Year 1** | **Year 2** | **Year 3** | **Year 4** | **Year 5** |
| --- | --- | --- | --- | --- | --- |
| **<350/µL** | | | | | |
| Initiating care due to previous ineligibility | - | - | - | - | - |
| Newly linked to care | 84,000 | 84,000 | 84,000 | 84,000 | 84,000 |
| **350-500/µL** | | | | | |
| Initiating care due to previous ineligibility | 40,000 | 40,000 | 40,000 | - | - |
| Newly linked to care | 18,000 | 18,000 | 18,000 | 18,000 | 18,000 |
| **>500/µL** | | | | | |
| Initiating care due to previous ineligibility | 57,000 | 57,000 | 57,000 | - | - |
| Newly linked to care | 23,000 | 23,000 | 23,000 | 23,000 | 23,000 |
| **Total initiating care** | 222,000 | 222,000 | 222,000 | 125,000 | 125,000 |

**Table S3.** Two- and five-year budgetary impact of a DTG-based regimen across a range of annual costs.

|  | **Undiscounted program costs (% change relative to SOC)** | |
| --- | --- | --- |
| **Strategy** | 2-year program cost^*^  (2016 USD, millions) | 5-year program cost^*^  (2016 USD, millions) |
| EFV/TDF/3TC (*SOC*) | 139 | 590 |
| *Annual cost of DTG+TDF/3TC* |  |  |
| $75 | 120 (-14%) | 518 (-12%) |
| $102 (base case cost) | 137 (-1%) | 590 (-) |
| $105 | 139 (-) | 598 (+1%) |
| $110 | 142 (+2%) | 611 (+4%) |
| $120 | 148 (+6%) | 638 (+8%) |
| $130 | 154 (+11%) | 665 (+13%) |
| $140 | 160 (+15%) | 692 (+17%) |
| $150 | 167 (+20%) | 718 (+22%) |
| $200 | 198 (+42%) | 852 (+44%) |
| $250 | 229 (+65%) | 986 (+67%) |
| $300 | 260 (+87%) | 1,120 (+90%) |

**DTG:** dolutegravir. **SOC:** standard of care. **EFV:** efavirenz. **TDF:** tenofovir disoproxil fumarate. **3TC:** lamivudine. **USD:** US dollars.

^*^ Costs projected for cohorts of 444,000 and 916,000 ART-eligible patients initiating treatment over two and five years.

**Figure S1. Schematic diagram for calculating HIV transmissions over 5 years under *SOC* and *DTG*.**

The figure provides additional detail on how the number of 5-year HIV transmissions and costs averted due to reduced transmissions were calculated under *SOC* and *DTG*. The number of prevalently-infected patients that initiate ART every year, for five years, are reported under the column “Prevalent cohort initiating care” and are grouped by CD4 count. The number of patients in the prevalent cohort are reported in the first row of each CD4 group section (numbers are bold). The number of HIV infections transmitted by these prevalently-infected cohorts each year are reported across the subsequent rows. The colors of the cells correspond to the transmissions attributable to the year of ART initiation (e.g., the transmissions in the red color cells are attributable to the cohort initiating ART at Year 1 while transmissions in the orange color cells are attributable to the cohort initiating ART at Year 2). Total five-year costs of transmissions were calculated by determining the average time to entering HIV care as a combination of average time to infection and average time between infection and linkage to care. The latter was determined from the model, in which mean CD4 count at acute infection, 553/µL, was compared to off-ART CD4 rates of decline and mean CD4 count at presentation to care in India. **SOC:** standard of care. **DTG:** dolutegravir.

**Figure S2. Multi-way sensitivity analysis on the cost-effectiveness of *DTG* compared to *SOC* in India while simultaneously varying *DTG* cost, probability of virologic failure with *DTG*, and second-line ART cost.**

The figure reports changes in the incremental cost-effectiveness ratio (ICER) of *DTG* compared to *SOC* when simultaneously varying the annual cost of *DTG* and the monthly probability of virologic failure after 48 weeks on *DTG* at two alternative second-line ART costs ($98 and $318 per-person per year). The horizontal axis denotes the range of annual costs of *DTG*. The vertical axis denotes the range of monthly probabilities of late failure for those virologically suppressed on the DTG regimen. The black “X” marks the characteristics of the base case *DTG*. The colors of the cells represent ICER categorization, ranging from “not cost-effective” (i.e., *DTG* confers a greater number of life years than *SOC* but at an incremental cost per life-year that exceeds 1x the Indian *per capita* GDP, orange cells), “cost-effective” (i.e., *DTG* confers a greater number of life years than *SOC* at an incremental cost per life-year that is less than the national GDP *per capita*, light green and yellow cells), to “cost-saving” (i.e., *DTG* both costs less and confers a greater number of life-years than *SOC*, dark green cells). **DTG:** dolutegravir. **ART:** antiretroviral therapy. **USD:** US Dollars. **ICER:** incremental cost-effectiveness ratio. **GDP:** gross domestic product.

**Figure S1.**

| **SOC** | | | | | | | | |  |
| --- | --- | --- | --- | --- | --- | --- | --- | --- | --- |
| *CD4 group* | *Year of transmission* | | *Prevalent cohort initiating care (first row of each CD4 group,* ***bold*** *numbers)* | | | | | *Total number of 5-year transmissions by group* | *Total 5-year cost of transmissions*  *by group (millions)* |
|  |  |  | **Year 1** | **Year 2** | **Year 3** | **Year 4** | **Year 5** |  |  |
| <350/µL |  | | **84,000** | **84,000** | **84,000** | **84,000** | **84,000** |  |  |
|  | Number of transmissions | **Year 1** |  | 3,100 | 3,100 | 3,100 | 3,100 | 21,700 | $1.3 |
|  |  | **Year 2** |  |  | 1,600 | 1,600 | 1,600 |  |  |
|  |  | **Year 3** |  |  |  | 1,500 | 1,500 |  |  |
|  |  | **Year 4** |  |  |  |  | 1,500 |  |  |
| 350-500/µL |  | | **58,000** | **58,000** | **58,000** | **18,000** | **18,000** |  |  |
|  | Number of transmissions | **Year 1** |  | 2,000 | 2,000 | 2,000 | 600 | 14,600 | $1.0 |
|  |  | **Year 2** |  |  | 1,200 | 1,200 | 1,200 |  |  |
|  |  | **Year 3** |  |  |  | 1,400 | 1,400 |  |  |
|  |  | **Year 4** |  |  |  |  | 1,600 |  |  |
| >500/µL |  | | **80,000** | **80,000** | **80,000** | **23,000** | **23,000** |  |  |
|  | Number of transmissions | **Year 1** |  | 2,800 | 2,800 | 2,800 | 800 | 21,100 | $1.2 |
|  |  | **Year 2** |  |  | 1,800 | 1,800 | 1,800 |  |  |
|  |  | **Year 3** |  |  |  | 2,100 | 2,100 |  |  |
|  |  | **Year 4** |  |  |  |  | 2,300 |  |  |
| **Total transmissions/cost:** | | | | | | | | **57,400** | **$3.6** |

| **DTG** | | | | | | | | |  | |
| --- | --- | --- | --- | --- | --- | --- | --- | --- | --- | --- |
| *CD4 group* | *Year of transmission* | | *Prevalent cohort initiating care  (first row of each CD4 group,* ***bold*** *numbers)* | | | | | *Total number of 5-year transmissions by group* | *Total 5-year cost of transmissions by group (millions)* | |
|  |  |  | **Year 1** | **Year 2** | **Year 3** | **Year 4** | **Year 5** |  |  |  |
| <350/µL |  | | **84,000** | **84,000** | **84,000** | **84,000** | **84,000** |  |  |  |
|  | Number of transmissions | **Year 1** |  | 2,500 | 2,500 | 2,500 | 2,500 | 16,400 | $1.1 |  |
|  |  | **Year 2** |  |  | 1,000 | 1,000 | 1,000 |  |  |  |
|  |  | **Year 3** |  |  |  | 1,100 | 1,100 |  |  |  |
|  |  | **Year 4** |  |  |  |  | 1,200 |  |  |  |
| 350-500/µL |  | | **58,000** | **58,000** | **58,000** | **18,000** | **18,000** |  |  |  |
|  | Number of transmissions | **Year 1** |  | 1,700 | 1,700 | 1,700 | 500 | 11,700 | $0.7 |  |
|  |  | **Year 2** |  |  | 900 | 900 | 900 |  |  |  |
|  |  | **Year 3** |  |  |  | 1,100 | 1,100 |  |  |  |
|  |  | **Year 4** |  |  |  |  | 1,200 |  |  |  |
| >500/µL |  | | **80,000** | **80,000** | **80,000** | **23,000** | **23,000** |  |  |  |
|  | Number of transmissions | **Year 1** |  | 2,400 | 2,400 | 2,400 | 700 | 16,300 | $1.0 |  |
|  |  | **Year 2** |  |  | 1,200 | 1,200 | 1,200 |  |  |  |
|  |  | **Year 3** |  |  |  | 1,500 | 1,500 |  |  |  |
|  |  | **Year 4** |  |  |  |  | 1,800 |  |  |  |
| **Total transmissions/cost:** | | | | | | | | **44,400** | **$2.8** | |
| **Transmissions/cost averted by *DTG* (vs. *SOC*):** | | | | | | | | **13,000** | **$0.8** | |

**Figure S2.**
